# Green synthesis of zinc oxide nanoparticles using ethanolic leaf extract of *Olea europaea* and its *in vitro* evaluation on MDA-MB-231 cancer cell lines, antibacterial and antioxidant activities

**DOI:** 10.1371/journal.pone.0339400

**Published:** 2025-12-29

**Authors:** Ismaila Ceesay, Pwadubashiyi Coston Pwavodi, Ridwan Olanrewaju Shittu, Huzaifa Umar

**Affiliations:** 1 Faculty of Engineering, Department of Bioengineering, Cyprus International University, Haspolat, Nicosia, Northern Cyprus Mersin 10, Türkiye; 2 Faculty of Engineering, Department of Biomedical/Medical Engineering/Bioengineering, Cyprus International University, Haspolat Nicosia, Northern Cyprus Mersin 10, Türkiye; 3 Operational Research Center in Healthcare, Near East University, TRNC, Mersin 10, Türkiye, Nicosia, Northern Cyprus Mersin 10, Türkiye; Cairo University, Faculty of Science, EGYPT

## Abstract

There are about 1.4 million cases of breast cancer in women that are diagnosed annually worldwide. Treatments to downregulate these tumor cells include surgery, immunotherapy, and chemotherapy. This study evaluates the synthesis of zinc oxide nanoparticles (ZnONPs) and *Olea europaea* (*O. europaea*) and its characterization, antioxidant, antimicrobial, and cytotoxic effects. The Gas Chromatography-Mass Spectrometry analysis indicated bioactive compounds that are present in *O. europaea,* such as Apigenin, Oleoside, Hydroxytyrosol, Rutin, Oleuropein aglycone, Tyrosol, and Oleuropein. The Ultraviolet visible spectroscopy analysis indicated the spectrum with peaks of 238 nm, 282 nm, and 313 nm. The zeta sizing analysis showed a size of 86 nm with a charge of −12.64 mV, X-RAY Diffraction (XRD), which shows the crystalline structure of the material, and the Fourier Transform Infrared Spectrophotometer (FTIR), indicating the chemical bond, molecular composition, and the functional group. Scanning Electron Microscopy (SEM) and SEM-EDS (Energy Dispersive X-ray analysis were done to determine surface morphology and elemental composition. An antioxidant activity assay was performed using diphenylpicryl hydrazine (DPPH) for free radical scavenging activity. The antibacterial inhibition assay was performed, and the results obtained were gentamycin 42.55 mm, *Pseudomonas aeruginosa (P. aeruginosa)* 29.22 mm, *Escherichia coli (E. coli)* 12.55 mm*, Staphylococcus aureus (S. aureus)* 16.40 mm*, and Bacillus cereus (B. cereus)* 8.44 mm. Cytotoxicity assay was performed on Dulbecco’s Modified Eagle Medium DMEM-grown MDA-MB-231 cells with varying dosage concentrations. The result showed that after 24 hours of treatment, cells were reduced to 60%, and after 48 hours of incubation, there was a 47% effect of ZnONPs. ZnO nanoparticle activity caused the MDA-MB-231 breast cancer cells to shrink, aggregate, deform, and proliferate more slowly. This research showed the medicinal potency of *O. europaea*.

## Introduction

Cancer is a complex, multifactorial disease in which normal cells are transformed into malignant cells, acquiring several properties, including enhanced proliferative and decreased apoptotic capabilities. In a healthy cell, the replication process is highly regulated, with several checkpoints that control the production of growth factors and the regulation of key signaling molecules, ensuring the normal proliferation of cells [1]. Breast cancer is the second leading cause of cancer deaths in women worldwide. According to 2018 data compiled by the World Health Organization, breast cancer is the most commonly encountered cancer in women (54% of the total) [2].

Breast cancer is a heterogeneous disease with distinct clinical behavior and molecular properties. In particular, estrogen receptor (ER)-positive and ER-negative cancers are the two most distinct subtypes. Since ER plays a central role in the crosstalk between different signaling pathways in breast cancer, the expression of this receptor is important for the behavior of breast cancer cells and is reflected in gene expression patterns of breast tumors [3].

Scientists concentrate on nutraceuticals as an emerging medication with fewer adverse effects to treat, and these minimize drug-related adverse effects and resistance. As science and technology advance, the demand for therapeutic herbal and non-herbal drugs grows. Thus, innovative, healthy nutrition substrates are abundant [4]. Many compounds originating from plant bio-products and medicinal plants have strong anti-cancer activities, such as Flavonoids, alkaloids, and polyphenols [4,5].

*O. europaea* leaf extracts have always been associated with health benefits and antibacterial, antiproliferative, and antioxidant activity [6]. Olive leaf antioxidants inhibit lipid peroxidation, prolonging food shelf life. Oleuropein, a bitter molecule that amounts to about 6–9% of leaf dry matter, has been studied for its health advantages [7].

Nanotechnology is a cutting-edge area that uses environmentally friendly methods for producing adaptable, non-toxic nanoparticles that are chemical-resistant and beneficial in biomedicine [8]. Nanomedicine, which uses nanostructured materials at a nanometre scale (1–100 nm) [9], improves chemical, physical, mechanical, and biological characteristics [10]. Researchers have provided numerous medical nanotechnology applications [11], highlighting its huge surface area, strong heat conductivity, catalytic reactivity, and chemical stability. Due to their surface charge, the lower uptake effectiveness of positively charged ZnONPs makes them safer [12], and they could also be used as biosensors, beauty products, and for the preservation of the environment, biology, and medicine [13].

The most common inorganic substance utilized in science and technology is ZnO [14]. Researchers are beginning to focus on green synthesis procedures since these approaches use a less dangerous method to generate nanoparticles [15]. Researchers have begun to manufacture eco-friendly and bio-inspired ZnONPs for antibacterial purposes [10]. ZnO is a viable green synthesis candidate owing to its non-toxicity, low cost, and antibacterial properties [16]. Therefore, the pharmacological activities of *O. europaea* are analyzed for its antioxidant, antibacterial, and cytotoxicity against breast cancer [17,18]. The green-synthesized ZnONPs possess surface-bound phytochemicals like oleuropein, hydroxytyrosol, and flavonoids from *O. europaea,* which causes them to display improved antioxidant activity when used in experiments. These compounds can donate their electrons or hydrogen atoms to neutralize free radicals and enhance reactive oxygen species (ROS) scavenging activity. Chemically synthesized ZnONPs are limited in antioxidant activity, except when they are functionalized, as they don’t have any surface bioactive compounds. Secondly, the green-synthesized ZnONPs exhibit dual antibacterial activity, as they increase the zones of inhibition due to both the action of ZnO and the phytochemicals present in the plant extract; however, a higher concentration of these green-synthesized ZnO nanoparticles is required to achieve the same effect as that of chemically synthesized ZnO nanoparticles [8,9]. The cytotoxicity activity of the ZnONPs on MDA-MB-231 cancer cell growth can be attributed to one or more apoptotic mechanisms that damage the mitochondria. cancer cell growth can be attributed to any of the following apoptotic mechanism that damages the mitochondria. First, the ZnONPs can produce ROS, which damage the cellular components and the mitochondria, leading to oxidative stress. The nanoparticles can also inhibit the respiratory chain’s activity and reduce ATP production, decreasing the cell’s energy production and affecting the mitochondria’s function. The nanoparticles can also change the balance of pro-apoptotic and anti-apoptotic proteins in the mitochondria. This leads to the production of cytochrome c and activation of the caspase cascade that results in programmed cell death. Secondly is the apoptosis mechanism by Caspase activation. Zinc is important for the function of p53, which is a tumor suppressor gene that controls apoptosis. This is done by activating the Caspace-6 enzyme. The ZnONPs have an electrostatic property that helps selectively target cancer cells, making them effective against them. The activation of Caspase that leads to the apoptosis of cancer cells can also occur by the formation of a complex combining cytochrome c, apoptotic protease activating factor, and pro-caspase 9, which triggers the expression and activity of caspase 3 and 7 that leads to cell death. Thirdly, apoptosis occurs as a result of the modulation of intracellular signaling pathways when Zn ions enter the cell through ion channels, which suppresses the activity of Bcl-2 markers and induces the expression of Bak/Bax to promote permeability. The anticancer agents invading the cancer cells could damage the electron transport chain, releasing ROS intracellularly, leading to apoptosis by the zinc ions dissolved within the cell. Lastly, apoptosis results from DNA damage and mitotic death caused by the overproduction of ROS, which also triggers autophagic and mitophagic cell death that could change gene expression. ZnONPs can affect the expression of Bcl-2 and other apoptotic genes, which will cause damage to the DNA and eventually cell death [19].

This study presents the anti-cancer activity of ZnONPs obtained from an *in vitro* study using MDA-MB-231 breast cancer cell lines, due to the complex mixture of bioactive compounds in *O. europaea*. The study suggested that *O. europaea* synthesized with ZnO might activate different signaling events and more effectively promote programmed cell death processes by targeting various metabolic susceptibilities in cancer cells. Consequently, we have demonstrated that ZnONPs treatment leads to cell death in all the cell models of cells used in the study. Furthermore, the utility of *O. europaea,* ZnONPs, and ZnO regarding antimicrobial and antioxidant characteristics was analyzed. However, after reviewing literature published in the past 10 years, we found that our work on ZnONPs’ impact on the MDA-MB-231 breast cancer cells is among the recent reports on the study (Table 1).

**Table 1:** Showing previous works about *O. europaea* Plant Parts and the type of cell lines used.

*O. europaea* plant	Part	Extraction Solvent	Cancer/Bacterial Subtype	*In vitro* or *Invivo*	Effects	Reference
***O. europaea***	Leave	H_2_O	MCF-7&MDA-MB 231	*In vitro*	The O2 has shown more biological activity. No more cytotoxicity effect on breast cancer MDA-MB-231 & MCF-7	[20]
**Olive Oil**	Olive Oil	cyclohexane and extraction with MeOH/H_2_O (80:20)	MCF-7&MDA-MB 231	*Invivo* *In vitro*	Enables cytotoxic/antiproliferative efficacy in both MCF-7 & MDA-MB-231	[21]
**Olive Leave**	leave	water	HL-60 leukemic cell	*In vitro*	Indicates high cytotoxicity on HL-60 leukemic cells	[22]
**Olive leaves**	leave	water	Tumor Cell	*In vitro*	OLEO reduced melanoma cell proliferation and motility	[23]
**Olive leaves**	leave	ethanol	S. aureus	*In vitro*	OL doesn’t indicate any effect despite crushing	[7]
***O. europaea***	leave	chloroform, n-butanol, and water	Mice	*Invivo*	The crude extract significantly reduced parasitemia and showed an antimalarial effect.	[24]
***O. europaea***	leave	(Ethyl alcohol, diethyl ether, acetone, and water	Both Gram Positive and Gram Negative	*In vitro*	Indicated a largely effect against gram-positive than gram-negative bacteria	[25]
***O. europaea***	leaves	Water, Ethanol	Both Gram-positive and Gram-negative MDA-MB-231 breast cancer cell lines	*In vitro*	Indicated effect against gram-positive and gram-negative bacteria. Indicated cytotoxic efficacy against the cancer cell lines.	This Study

## Materials and methods

### Materials

GCMS-QP2010 Plus System (Shimadzu, Japan), 35 mm cell culture dishes (Thermo Scientific Nunc Cell Culture Dishes; Thermo Scientific; USA), and a UV–visible spectrum were evaluated using a UV–visible spectrophotometer (Shimadzu UV-2450), Dulbecco’s Modified Eagle Medium (DMEM) (Gibco by Life Technologies™, USA), 1,1-diphenyl-2-picrylhyorazyl (DPPH), and an inverted microscope (Leica DFC295).

### Identification and collection

**Fig 1:**
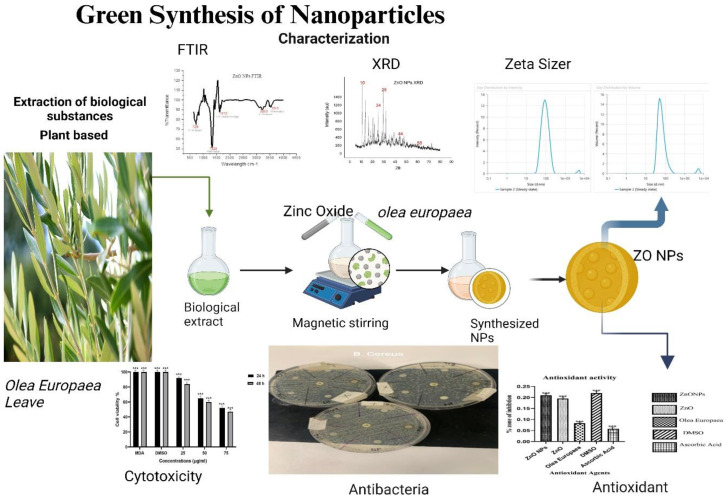
Shows the procedure that was used for all the assays that were carried out.

The fresh *O. europaea* was collected in Lefkosa Kucuk Kaymakli (Northern Cyprus) with geographical coordinates of 35.2032 North and 33.3674 East. Normally, the best season to collect the leaves is from September to January, but we collected in October, when the leaves and fruits are green. Its botanical feature was identified as *O. europaea* by a pharmacognosist at the Faculty of Pharmacy, Cyprus International University, and was given a voucher number (CIU/PHAR/OLEA/001). It was prepared and deposited at the Faculty of Pharmacy, Cyprus International University, for future reference (Fig 1).

### Ethics and consent approval

Local ethical permissions were obtained from the local authorities before collecting the plant used for the study.

### Preparation and extraction

Extracts of *O. europaea* were produced using several filtering processes: lyophilization, formulation to a certain stock concentration, and sterilization. The cleaned leaves were macerated in water (Fig 1). Subsequently, the heterogeneous mixture was passed through a nylon mesh to exclude larger fibers. The filtrate was centrifuged at 5000 rpm at ambient temperature for 6 minutes. Subsequently, a Buchner funnel filters the supernatant before lyophilization, resulting in a fine powder of about 40 g. The yield from lyophilization was 43% w/w; the blended composition was filtered using Whatman No. 1 filter paper, removing minor fibrous impurities from the filtrate. Subsequently, the powder is reconstituted to a designated stock concentration using sterile 1X Phosphate Buffer Saline (PBS), and the *O. europaea* is further diluted before application to breast cancer cells in a medium [26].

### Synthesis of ZnO nanoparticles

Fresh *O. europaea* were collected and washed thoroughly with tap water, then rinsed with distilled water to remove particles of sand and dirt. The leaves were air-dried at room temperature for 5–7 days to preserve the bioactive compounds in them. Dried leaves were ground to a fine powder using a clean blender. 10 g of powdered leaves were boiled in 100 mL of distilled water for 30 minutes at ~80°C. The mixture was allowed to cool and then filtered using Whatman No. 1 filter paper. The filtrate was stored at 4°C and used within 48 hours to preserve phytochemical activity. 0.1 M of zinc nitrate hexahydrate was dissolved in 100 mL of distilled water, placed on a magnetic stirrer, and stirred constantly. 2.5 mL of the extract was added to the zinc nitrate solution under continuous magnetic stirring at 60°C for 5 hours for complex formation. Finally, 5 mL of 0.5 M NaOH was added to the solution while stirring, and the pH was adjusted between 10 and 11. A color change was observed from pale green to light yellow, indicating ZnO NP formation. The mixture was stored overnight at room temperature. All the mixtures were placed in an oven at 50°C for 72 hours to allow for complete drying and formation of the solid ZnO nanoparticle [27].

### Qualitative test for the presence/absence of phytochemicals of *O. europaea*

The phytochemical screening of the *O. europaea* plant extract was carried out according to the procedures described by [28].

### Gas Chromatograph Mass Spectrometry GC-MS

The phytochemical screening was assessed on a GC-MS QP2010 plus, a product of Shimadzu, and a column type of Teknokroma TRB at a rate of 5°C/min at an initial temperature of 50°C and a final temperature of 280°C with a 2-minute holding time. The total duration of the time that was taken was 31 minutes, using helium as a carrier gas. The mobile phase flow rate was programmed at 1 mL/min, and the ionizing voltage was adjusted relative to tuning. The mass scan ranges from 35 to 1000 amu at 70 eV using an injection volume of 1 µL. The spectra of the compounds in the extract were compared with the already interpreted and known components found in the GC-MS database.

### Characterization

To provide a comprehensive description of the ZnONPs that had been synthesized, a wide variety of spectroscopic and microscopic techniques were used. The UV-visible spectrum was recorded between 200 and 800 nm using a Shimadzu UV-2450 UV-visible spectrophotometer. The spectrum was measured using the UV–visible spectrum. The Malvern Zeta sizer Nano ZS90 was used to estimate the hydrodynamic (Z-average) size of the ZnONPs that were created, and the Malvern ZS Nano software was used to capture the data. The Zeta sizer equipment was used to determine the size of the ZnONPs. To assess the composition of the NPs, an infrared Fourier transform (FTIR) study was carried out on them using a Shimadzu FT-IR Prestige-21 Model Fourier Transform Spectrometer with a frequency range of 4,000–500 cm⁻¹. The crystal structure was examined using a Rigaku ZSX Primus II X-ray diffractometer. Additionally, the crystallinity test was done using an Advanced XRD instrument with Cu Kα radiation (λ = 1.5406 Å nm wavelength), and the formation of ZnONPs and the structure and composition were done using Ni filter power supply 40 kV/20 mA in the range of 3° ≤ 2θ ≤ 50°. Scanning Electron Microscope (SEM) and SEM-Energy Dispersive X-ray Analysis (SEM-EDS) were also performed to characterize the samples that have been synthesized by coating the samples with platinum [29].

### The antioxidant activity assay

The DPPH (1,1-diphenyl-2-picrylhydrazyl) assay was used to calculate the antioxidant effectiveness of the sample. The DPPH reagent and sample solution were prepared. The sample of *O. europaea*, ZnONPs, and Zn was made by 6 series of concentrations: 100, 50, 25, 12.5, 6.25, and 3.5 mg/mL, with quercetin standard in the same range of 6 series, respectively, then observed absorption in the wavelength range 517 nm using a UV-2450 spectrophotometer (SHIMADZU). Each 1 mL of the sample was mixed with 3 mL of DPPH, which was then reduced to DPPH-H. The maximum wavelength of the DPPH solution was determined by creating a reference solution that combined 4 mL of the prepared DPPH solution with ethanol and shaking it until homogeneous. A decrease in absorbance indicated the scavenging activity of the antioxidant compound in the sample, measured in terms of its H-donating ability. The incubation was done in the dark to avoid the degradation of the DPPH for 30 minutes at a room temperature of 25˚C. The uptake test was performed at the maximum wavelength, of the DPPH value of antioxidant activity is expressed as [30]


$$\%\;\text{DPPH}\;\text{Activity}=\frac{Absorbance\;Control-Absorbance\;Sample}{Absorbance\;Control}\times \text{1}\text{0}\text{0}\ldots$$ (1)

The scavenging activity of the samples was assessed using the percentage scavenging activity at six different concentrations. The effective concentration, which is the concentration (mg/mL) of the *O. europaea* needed to remove 50% of the radicals from the samples, was determined using the IC_50_ format, which is the value defined as the substrate concentration that causes the loss of 50% of the activity of DPPH, against the percentages of inhibition according to different dilutions of the extracts used for the analysis.

### Antimicrobial assay

The Kirby-Bauer disc diffusion method was used for the antimicrobial activities of concentration for *O. europaea*, ZnONPs, and Zn discs diffused with 10 μL and 30% DMSO as the negative control, and gentamycin (10 mg/mL) was the positive control against six bacterial cells, which are both Gram-positive (*S. aureus, E. faecalis, B. cereus*) and Gram-negative (*E. coli, P. aeruginosa, L. pneumophila*) bacteria that were available, and these allowed for the assessment of the spectrum of activities [31–34]. *B. cereus, Legionella, P. aeruginosa, S. aureus, E. faecalis,* and *E. coli*. This procedure was carried out in the biosafety cabinet of the pharmacy laboratory at Cyprus International University. The 100 µL of each prepared bacterial inoculum*—S. aureus, Legionella, P. aeruginosa, E. coli, B. cereus, and E. faecalis—was spread evenly on the labeled agar plates corresponding to each bacterial pathogen using a sterile swab and* incubated for 24 hours. After which, the *O. europaea*, ZnONPs, Zn, and the DMSO negative control were grown overnight in Nutrient Agar media, and then the zone of inhibition was determined by taking the diameter measurement of maximum inhibition by the bacteria to ascertain the antimicrobial activity of ZnONPs, Zn, and *O. europaea,* and with the DMSO negative control against the six microorganisms [30]. *L. pneumophila, S. aureus, E. faecalis, E. coli, P. aeruginosa,* and *B. cereus* were obtained from a microbiology laboratory at Cyprus International University.

### Minimal Inhibitory Concentration (MIC) and Minimal Bactericidal Concentration (MBC)

The antimicrobial efficacy of the ZnO nanoparticles (NPs), *O. europaea, and* ZnO was assessed by determining the minimal inhibitory concentration (MIC) and the minimal bactericidal concentration (MBC) using the standard microdilution method (CLSI). The bacteria were cultured in Mueller-Hinton (MH) broth until they reached the mid-log growth phase, and the initial suspension was adjusted to achieve a final density of 3 × 105 colony-forming units per milliliter (CFU/mL). 10 µL of several strains of diluted bacterial suspensions was added to the corresponding wells in a 96-well plate. Each sample was serially diluted with ZnONPs, ZnO, and *O. europaea* from 100 mg/mL to 1.5. The plates treated with a small amount of a substance to stimulate the growth of microorganisms were placed in a controlled environment at 37°C for 24 hours. The MIC was determined to be the lowest concentration of ZnO nanoparticles (NPs), which prevents observable growth. The MBC was determined by inoculating 10 µL of the samples from wells onto MH agar plates, which were then incubated at 37°C for 24 hours. When no growth was observed on the MH agar after incubation, it was determined to be the MBC.

### Cell culture

The breast cancer cell line MDA-MB-231 was obtained from Imperial College London, United Kingdom, and its use was approved by the ethical committee of the Biotechnology Research Center (BRCEC2011−01). Cells were grown in DMEM supplemented with 10% l-glutamine, 2 mM glutamine, and penicillin/streptomycin (Gibco by Life Technology, Carlsbad, CA). The cells were continuously kept in a humid chamber at 37°C and 5% CO_2_; analytical grades were utilized for all the substances used in the cell culture.

### Cytotoxicity assay

A trypan blue dye exclusion test assessed cytotoxicity in MDA-MB-231 cells. Three 35 mm tissue culture plates were used for each condition of the experiment, and every plate contained 1 mL of DMEM growth media and 3 × 104 cells. The cells were then left to incubate overnight to settle. After giving the cells various doses (25, 50, and 75 μL), the media in the plates were withdrawn 24 and 48 hours later, and diluted trypan blue was added (250 μL of trypan blue dye formulation and 800 μL of medium). After 15 minutes of incubation in the dark, the diluted trypan blue was removed, and 30 fields of view and imaging software were used to count the number of cells in each area recorded under a microscope set at a magnification of 10, called Leigen, which shows blue-coloured dead cells in contrast to transparent live cells. Three different dishes were used for this process. The average of three measurements multiplied by thirty [29].


$$\%Cell\;viability=\frac{number\;of\;live\;Cells\;}{total\;number\;of\;cells}$$ (2)

### Statistical data analysis

All experiments were performed at least three times independently, and IL, USA, was used for statistical analysis. The study used the SPSS 17.0 software (SPSS Inc., Chicago). Multiple group comparisons were analyzed with one-way ANOVA. The data analyzed were expressed as mean ± standard deviation. The differences were considered statistically significant when p < 0.001 and p < 0.0001.

## Results and discussion

### Synthesis of the ZnONPs with *O. europaea*

**Fig 2:**
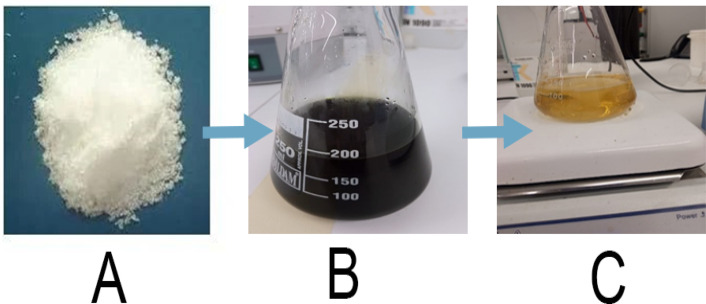
The green synthesis process of ZnONPs from *O. europaea.*

The preparation and green synthesis of ZnO nanoparticles (Fig 2) from *O. europaea* indicated a change in the color of the zinc nitrate solution after adding *O. europaea*. This color change suggested the successful formation of the ZnONPs. (A): colorless zinc nitrate precursor; (B): *O. europaea*, showing a dark brown (C): pale yellow formation, indicating the formation of ZnONPs.

### The Phytochemical evaluation of *O. europaea*

The phytochemical assay performed on *O. europaea* indicated the presence of tannins, saponins, alkaloids, steroids, and flavonoids (Table 2), and a similar result has been confirmed for its anti-inflammatory, antioxidant, and antinociceptive properties [35]. The analysis was carried out on *O. europaea*. Alkaloids, flavonoids, and proteins were all found in chemical tests [36].

**Table 2:** Phytochemical test for *O. europaea* showing the presence or absence of compounds.

Test	Observation	Result
Alkaloids Drangendroff Mayers Hager’s	Orange-red Yellowish yellow	+ve
Saponins Foaming test	Formation of foam	+ve
Tannins Lead acetate Ferric chloride	Gelatinous precipitate Greenish black	+ve
Flavonoid NaOH HCL test	Yellow colorless	+ve
Steroids	greenish	+ve
Anthraquinones	No change	-ve

+ve=present, -ve=absent

### GC-MS – Analysis of the compounds present in *O. europaea*

**Fig 3:**
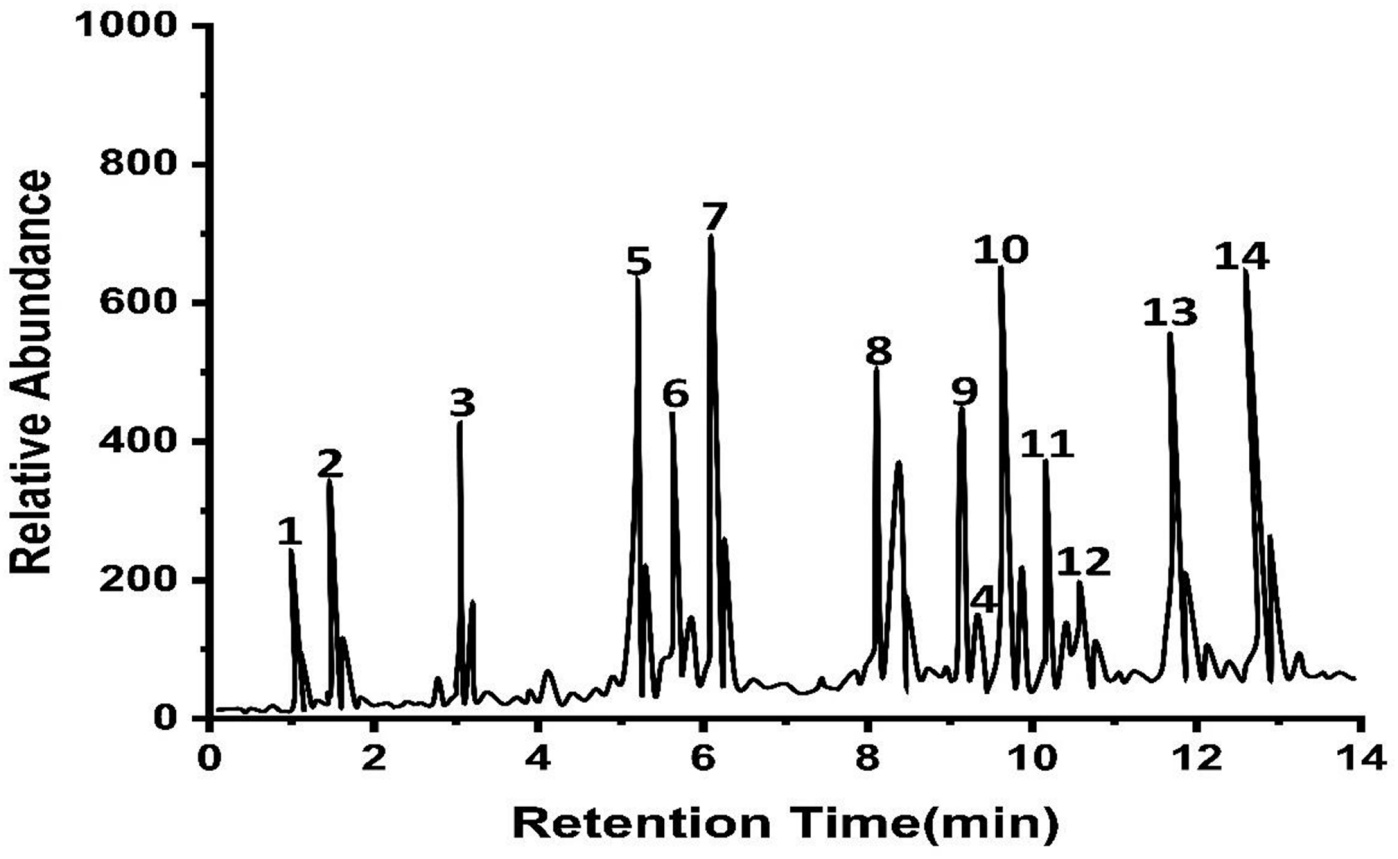
Illustrates the chromatogram derived from the GC-MS analysis of *O. europaea*, exhibiting several peaks according to the concentration of the chemicals. The Y-axis denotes relative abundance, while the X-axis indicates the elution duration of each component from the column. The applied rate was 5°C/min, commencing at a starting temperature of 50°C and concluding at a final temperature of 280°C, with a hold duration of 2 minutes. The overall time was 31 minutes, utilizing helium as the carrier gas.

The GC-MS analysis for identifying bioactive compounds in *O. europaea’s* ethanolic plant extract suggested the presence of several chemical components (Table 3). Polyphenols, flavonoids, and triterpenes are a few of the substances discovered. Fourteen bioactive compounds were present in the ethanolic extract of *O. europaea* after the GC-MS analysis was performed. The data library built into the GC-MS was used to analyze them (Fig 3).

**Table 3:** Shows the bioactive compounds present in *O. europaea* and the total ion chromatogram.

Number	Names	Present	Time Retention (min)	Formula	g/mole
1	Apigenin	+	1.33	C_15_H_9_O_5_	270.24
2	Oleoside	+	1.70	C_16_H_21_O_11_	374.36
3	Elenolic acid glucoside isomer 1	+	3.13	C_17_H_23_O_11_	404.38
4	Hydroxytyrosol	+	9.2	C_14_H_19_O_8_	154.16
5	Rutin	+	5.50	C_27_H_29_O_16_	610.52
6	Luteolin 7-O-glucoside	+	5.63	C_21_H_19_O_11_	448.38
7	Oleuropein diglucoside isomer 1	+	6.06	C_31_H_41_O_18_	694.64
8	Oleuropein aglycone	+	8.09	C_16_H_25_O_10_	378.36
9	Chrysoeriol-7-O-glucoside	+	9.01	C_22_H_21_O_11_	460.38
10	Verbascoside	+	9.58	C_29_H_36_O_15_	624.59
11	Oleoside	+	10.03	C_13_H_18_O9	318.27
12	Tyrosol	+	10.5	C_8_H_10_O_2_	138.16
13	Oleuropein	+	11.80	C_25_H_31_O_13_	540.52
14	Oleuropein isomer	+	12.87	C_25_H_31_O_13_	545.52

### UV-Vis Spectroscopy analysis of *O. europaea*

**Fig 4:**
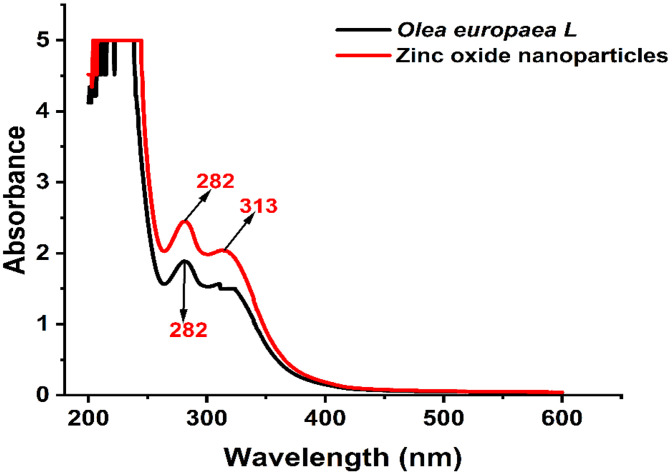
UV-visible spectrophotometry showing the overlap of *O. europaea* and ZnONPs, with their peaks indicated by the wavelength.

The UV-Vis spectroscopy reveals peaks indicating compounds that are present in the *O. europaea,* as shown in Fig 4, the overlap of ZnONPs and *O. europaea*, with different peaks. *O. europaea* extract was used as a bio-reductant to produce ZnONPs quickly. The noticeable color shift in (Fig 2) suggests the synthesis of ZnONPs from a ZnO solution. UV-vis spectroscopy was used to investigate this process [37]. The findings of this research demonstrate that the absorption wavelength of (282–313) for the synthesized *O. europaea* peak was at 313 nm, which is consistent with the ZnONPs’ absorption range of 300–320 nm [38]. However, Fuentes et al. claim that apigenin and luteolin, flavonoids included in the *O. europaea* phenolic component, explain the absorption at around 310–320. The use of various biological substrates with various compositions, the amount of ZnO, the reaction time, the pH, and the temperature significantly impact the particles’ sizes and morphologies. After considering the impact of the numerous synthesis requirements, including temperature, biological material zinc concentration, and reaction time, the UV–Vis Spectra of *O. europaea* showed peaks at 238, 285, and 313nm. This is similar to the result of [38].

### Zeta analysis of ZnONPs

**Fig 5:**
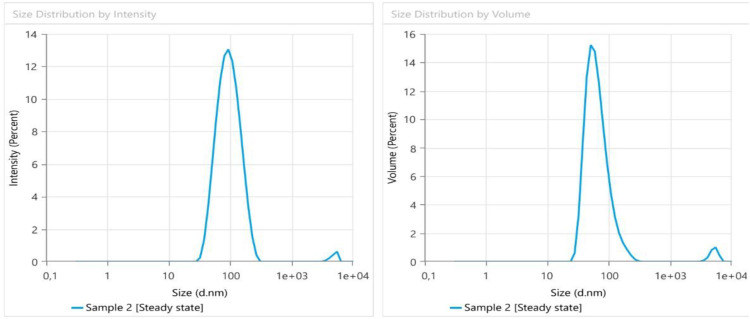
The zeta potential shows the average size of the ZnONPs.

A zeta sizer machine was used for analysis, and the particle size and potential charge distribution of ZnONPs were determined using an extract of *O. europaea.* After sonication for 5 minutes, the ZnONPs’ zeta potential and the particle size distribution (Fig 5) were measured using the Malvern Zeta Analyzer indicated below. This work investigated the size, homogeneity, and stability of ZnO-NPs using the DLS approach. The findings were shown as a size distribution depending on quantity and intensity. The degree of homogeneity increases with decreasing average size, and the degree of size scattering of the nanoparticles is indicated by the divergence of the PDI (polydispersity index) from the target value. ZnONPs had a size of 86 nm, and the nanoparticles’ zeta potential was also investigated since it is a physicochemical characteristic that may be utilized to estimate the surface charge and foretell the long-term durability of nanoparticles [39]. ZnO-NPs’ zeta potential value is 12.6 mV, suggesting reasonably stable particles (Fig 5). The Z-potential measurements determined on the ZnONPs produced by green synthesis showed that they are highly anionic. These findings attest to and validate the nanoparticles’ ability to disperse. The binding affinity of the extract chemicals with the nanoparticles, which imparts stability and lowers the possibility for aggregation of the particles, is attributable to the negative surface charge. Additionally, earlier research has shown that the biogenic ZnO-NPs synthesized by L. plantarum had a zeta potential value of 15.3 mV [40].

### FTIR analysis of ZnONPs

**Fig 6:**
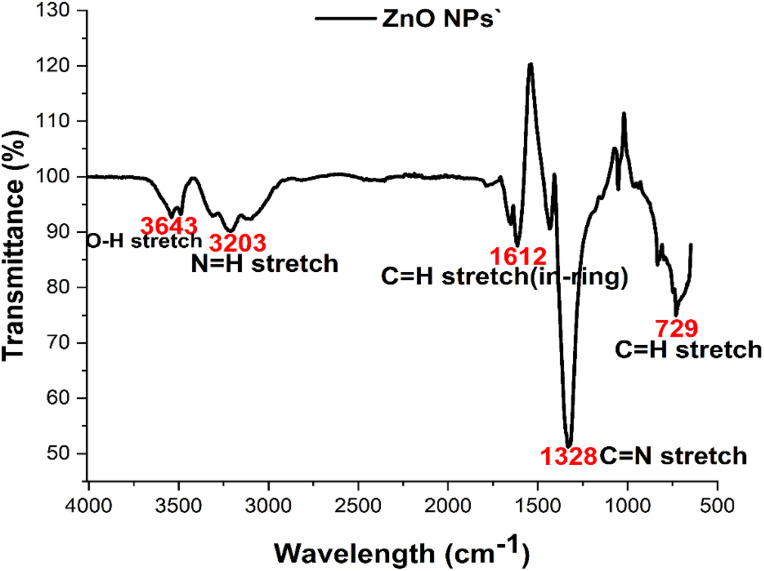
FTIR analysis spectrum of ZnONPs showing the different functional groups.

The analysis of the ZnONPs shows different functional groups and absorption peaks, which are attributed to both primary and secondary protein and OH-stretching, as indicated. FTIR was used to learn about the kinds of proteins involved in reducing and forming ZnO nanoparticles. The FTIR spectra of ZnO nanoparticles made from *O. europaea* leaf extract are shown in (Fig 6 and Table 4) [38]. The FTIR study found the bands at 729 cm⁻¹, 1328 cm⁻¹, 1612 cm^-1^,3203 cm^-1^, and 3543 cm^-1^. The range that was taken for the FTIR is between 500 cm⁻¹ and 4000 cm^-1^. The C-N stretching wave was given the band at 1328 cm^-1^, O-H wave 3543 cm⁻¹. C-H was given 729 cm^-1^, C = O, and N-H was given credit for the stretching vibrations at 1612 cm^-1^, and 3203 cm^-1^, shown in Fig 6. That bioreduction processes involve alkaloids, flavonoids, tannins, steroids, and saponin groups. In the leaf extract that was used to find out if it had steroids, it was thought that flavonoids, glycosides, proteins, and phenols caused bioreduction. The result was similar to the findings of [41].

**Table 4:** Shows the result of the FTIR analysis of ZnONPs.

Wavenumber (cm^-1^)	Functional group
3543	O-H stretch
3203	N-H stretch
1612	C = O stretch
1328	C-N stretch
729	C-H stretch

### FTIR analysis of *O. europaea*

The analysis of the ZnONPs shows different functional groups and absorption peaks, which are attributed to both primary and secondary and protein and OH-stretching, as indicated (Table 4) below. The analysis of the ZnONPs shows different functional groups and absorption peaks of percentage transmittance against the wavelength in cm⁻¹, which are attributed to primary, secondary, protein, and OH-stretching, as indicated in (Fig 6). The analysis of the ZnONPs shows different functional groups and absorption peaks attributed to primary, secondary, protein, and OH-stretching (Table 4).

**Fig 7:**
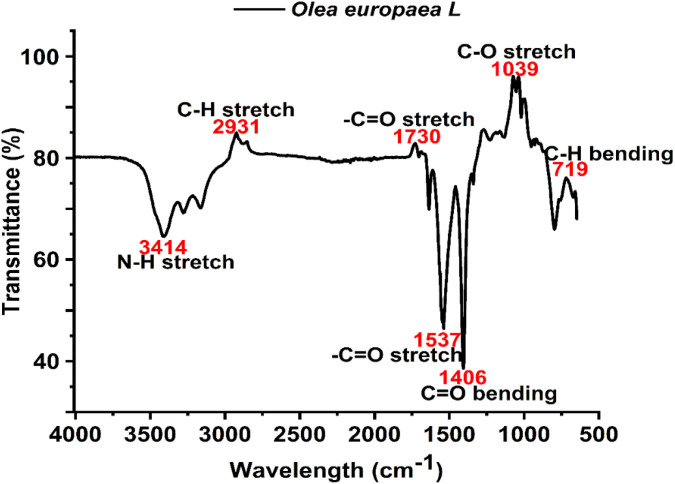
FTIR analysis of *O. europaea* showing different functional groups.

The analysis of the *O. europaea* shows different functional groups (Table 5) and absorption peaks, which are attributed to primary, secondary, protein, and OH-stretching (Fig 7), indicating percentage transmittance against the wavelength cm⁻¹.

**Table 5:** Shows the result of FTİR analysis for *O. europaea.*

Wavenumber (cm^-1^)	Functional group
3414	N-H stretch
2931	C-H stretch
1730	-C = O stretch
1537	-C = C stretch
1406	C = O stretch
1039	C-O stretch
719	C-H stretch

### XRD analysis

**Fig 8:**
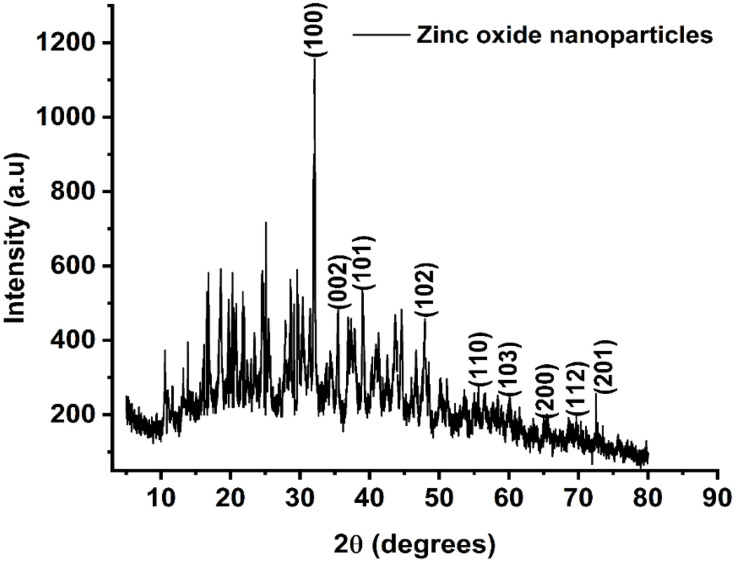
Illustrates the XRD of ZnONPs with the JCPDS number added on the plot for the nanoparticles synthesized.

XRD Results indicating different peaks of ZnONPs using significant functional groups are shown in Fig 8. It provides information on the crystal structure and size of the ZnONPs. The XRD patterns shown by ZnO nanoparticles produced from *O. europaea* extract were distinctive. The hexagonal phase of ZnO was found to be compatible with the peaks that were seen when XRD spectra were analyzed at certain levels shown in (Fig 8) for the ZnONPs and the ZnO with multiple Bragg reflections of 2θ value was seen, each with values of (100) at 31.7°, (002) at 34.4°, (101) at 36.2°, (102) at 47.5°, (110) at 56.6°, (103) at 62.8°, (200) at 66.4°, (112) at 68.0°, and (201) at 69.1° with JCPDS number of 36–1451. The lattice parameters from the peak positions are a = 3.25 Å and c = 5.21 Å. These labels confirm the formation of pure crystalline ZnO nanoparticles in the Wurtzite hexagonal phase without secondary phases, which often appear in biologically synthesized samples. The 2θ values are approximated from your pattern; the d-spacing and crystallite size are calculated using Bragg’s law and the Scherrer equation (using the (101) reflection) with a calculated average crystallite size of 25 nm (Table 6). The additional weak peaks or shoulder signals observed in the XRD result, such as 20.3°, 26.6°, 37.9°, 17.9°, 36.4°, 39.3°, 43.2°, 31.6°, 45.4°, 56.5°, 22°, 27°, and 43°, may be due to incomplete drying or synthesis at low temperature leading to hydroxide residue, atmospheric CO₂ absorption during the synthesis procedure, or contamination from glassware or substrates used in the process.

**Table 6:** Shows the result for 2θ, d-spacing, hkl indices, FWHM, and crystallite size (Scherrer calculation from the (101) peak).

2θ (°)	d-spacing (Å)	hkl	FWHM (°)	Crystallite size (nm)
31.7	2.82	(100)	0.30	28.4
34.4	2.60	(002)	0.32	27.2
36.2	2.47	(101)	0.35	25.0
47.5	1.91	(102)	0.38	23.1
56.6	1.62	(110)	0.40	22.4
62.9	1.47	(103)	0.42	21.3
66.4	1.41	(200)	0.45	20.0
67.9	1.38	(112)	0.46	19.8
69.1	1.36	(201)	0.47	19.3

### SEM-Energy dispersive x-ray analysis

**Fig 9:**
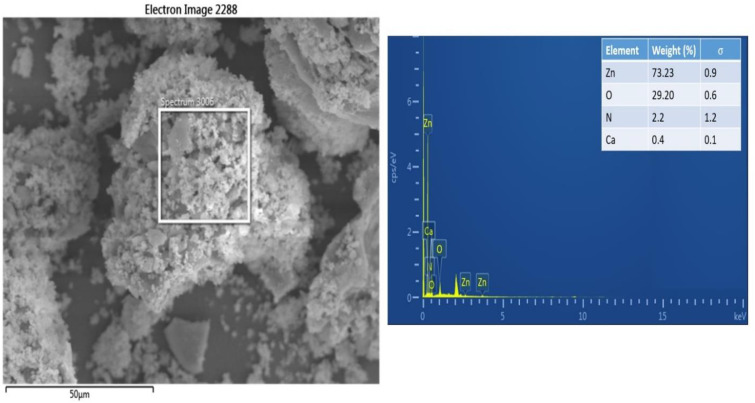
EDS Spectrum showed the purity of ZnONPs and the percentage weight distribution of Zn, O, N, and Ca in the ZnONPs sample.

The SEM-EDS analysis was carried out to determine the elemental quantitative and qualitative composition of the synthesized ZnONPs using the Oxford Instruments AZTEC EDS. The structural characterization of ZnONPs (Fig 9) gave information about the formation of ZnONPs. Metallic ZnO is suggested to be present in biosynthesized ZnONPs. The obtainable composition from the analysis was shown from the peaks, which were identified as Zinc (73.23%), Oxygen (29.20%), Nitrogen (2.2%) and Calcium (0.4%). The Zn was presented to have the highest absorption peaks. There were trace amounts of nitrogen and calcium, demonstrating that plant phytochemical groups are involved in reducing and capping synthesized ZnONPs [9].

### Scanning Electron Microscope (SEM)

**Fig 10:**
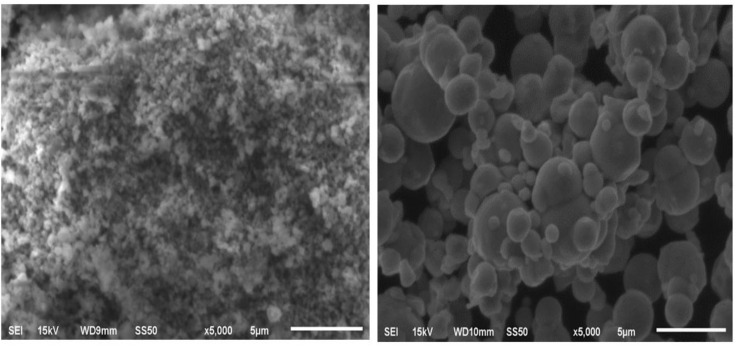
SEM image of ZnONPs synthesized using *O. europaea l*eaves.

The SEM analysis was carried out to visualize and determine the surface morphology and size of ZnONPs using the JEOL JSM 6335-F device. SEM images that were seen at different working distances demonstrated the presence of Zn ONPs formed with a spherical shape having smooth edges (Fig 10). The green synthesis of Zn ONPs from *O. europaea* extract appeared as self-aggregated in a close-packed periodic array of spherical, smooth-edged shapes and showed different particle sizes of the nanoparticles [9].

### Antioxidant assay

**Fig 11:**
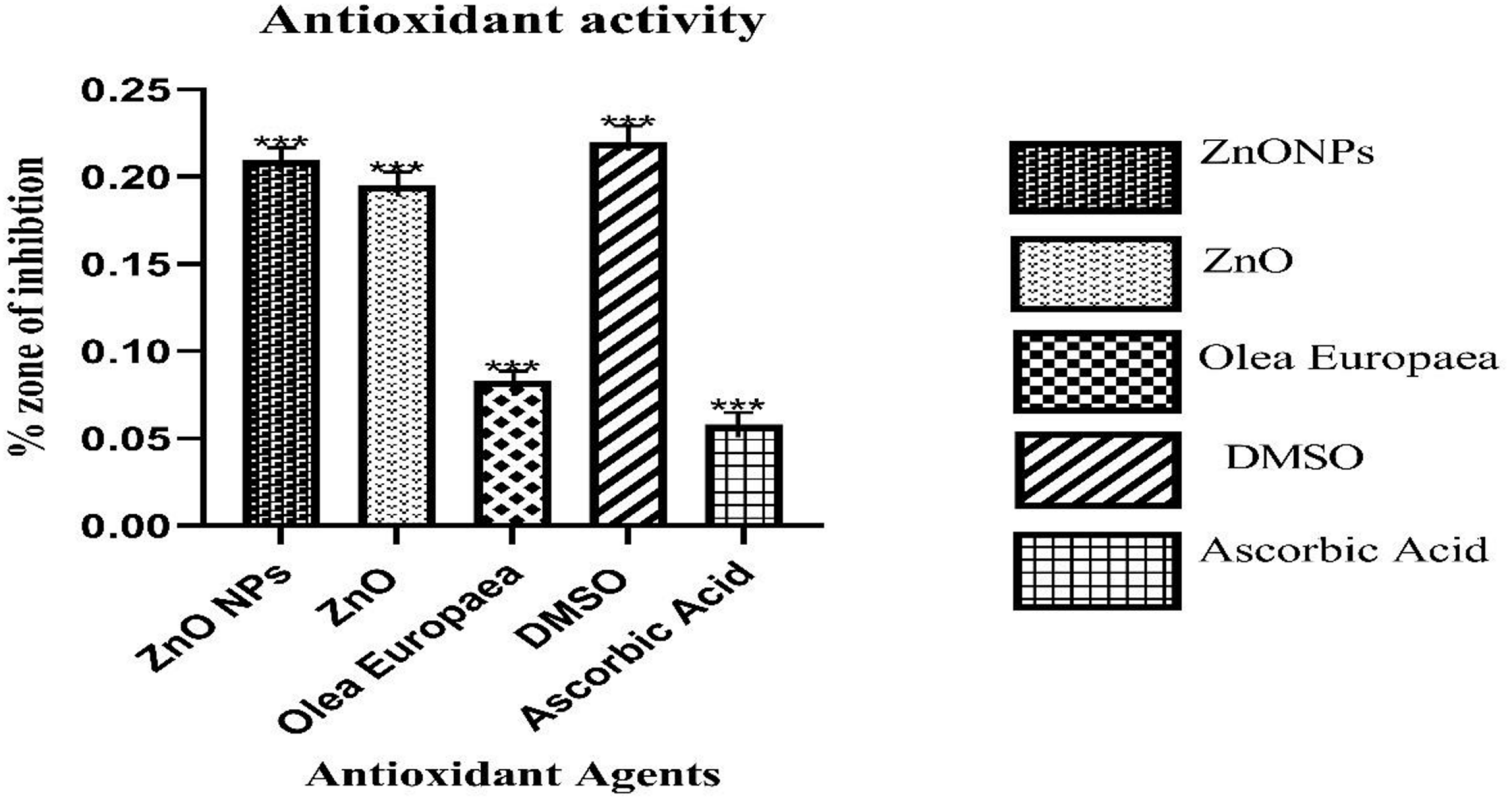
Shows DPPH Inhibition of ZnONPs, *O.E.L*, Zn, Ascorbic Acid, and DMSO.

DPPH is a stable free radical that measures a substance’s antioxidant activity. The ability of DPPH to get rid of free radicals was measured by how much its content dropped when antioxidative chemicals were added [42]. According to the findings, DMSO was more active, followed by ZnO nanoparticles, ZnO precursor, *O. europaea,* and ascorbic acid, with the following concentrations: 10 mg/mL, 5 mg/mL, 2.5 mg/mL, 1.56 mg/mL, and 0.625 mg/mL, respectively. The result (Fig 11) shows the graphical bar representation of the DPPH inhibition of various analyses: ZnONPs, ZnO precursor, *O. europaea*, DMSO, and ascorbic acid. In the concentration range of 100 to 1.56 μg/ml, the DPPH radical-scavenging activity of ZnONPs, Zn, *O. europaea*, ascorbic acid, and DMSO increased with the dose (Fig 11) [43]. The amount of discoloration showed how well the antioxidant leaf extract could get rid of free radicals. This study indicated that *O. europaea* has antioxidant activity [44]. ZnONPs and Zn exhibited small degrees of antioxidant activity when compared to the standard control, which might result from the method; hence, the size distribution and the stability have confirmed its antioxidant activity [45]. However, other research has found that Zn and ZnONPs have antioxidant activity, which was tested, and the plant extract served as a reducing agent during the manufacturing of nanoparticles [46]. These results were analyzed and contrasted with those obtained using ascorbic acid as the gold standard. The DPPH inhibitory activity of ZnONPs displayed scavenging activity results of 0.21 ± 0.0187, Zn 0.195 ± 0.032, *O. europaea* 0.083 ± 0.0063, DMSO 0.22 ± 0.023, and ascorbic acid 0.058 ± 0.0016, respectively.

### Antibacterial activity

**Fig 12:**
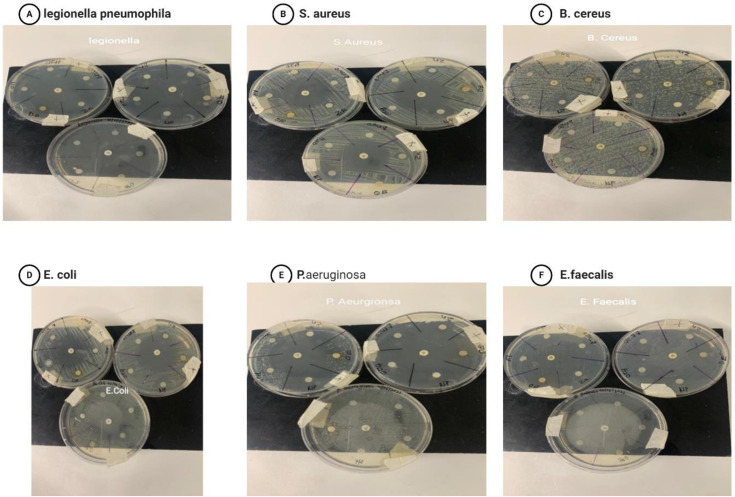
Zone of inhibition of the tested sample against (A) *Legionella pneumophila,(B) S.aureus,* (C) *B.cereus,* (D)*E. coli,* (E) *P. aeruginosa* (F) *E faecalis.*

**Fig 13:**
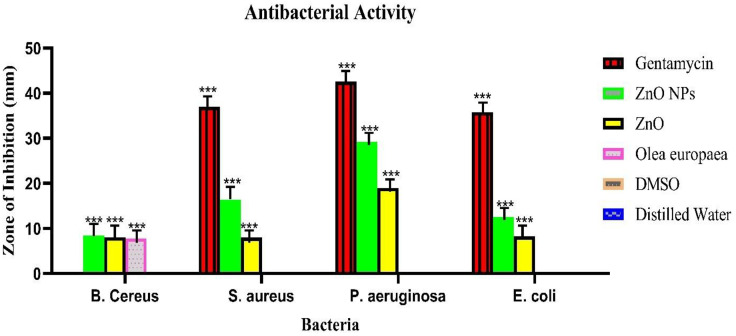
Disc diffusion results for antibacterial activity of *O. europaea*, ZnONPs, Zn, gentamycin, H₂O, and DMSO were obtained from (Table 7), indicating the zone of inhibition against *B. cereus, S. aureus*, *P. aeruginosa*, and *E. coli* with (mean ± SD mm). The values are expressed as mean ± SD values and analyzed by two-way analysis of variance (ANOVA). An asterisk (_***_) indicates significant value for treatments concerning control (P < 0.01).

The antibacterial activity of disc diffusion indicates that the test samples, including ZnONPs, Zn, and O. europaea, and the widely known drug gentamycin, were used (Fig 12, A-F) and (Fig 13) with a bar chart showing the zone of inhibition. The findings revealed that the conventional medication had the maximum activity, ranging from 42.55 to 35.77 to 36.96. In contrast, ZnONPs and Zn exhibited greater susceptibility to all the microorganisms tested than *O. europaea*. For ZnONPs, the activity varied from 29.22, 12.55, 16.40, and 8.44 mm, while for ZnO, it was between 18.99, 8.22, 7.99, and 8.0 mm. *O. europaea* findings were 7.77 mm only [36], indicating that it has less activity than other antibacterial agents, whereas in *E. faecalis* 0*, Legionella pneumophila* 0 [47,48]. However, we used distilled water and DMSO as negative controls and gentamycin as a positive control against bacterial pathogens: 12 A) *Legionella pneumophila*, (12B) *S. aureus*, (12C) *B. cereus,* (12D) *E. coli,* (12E) *P. aeruginosa,* and (12F) *E. faecalis* after 24 hours of incubation. Apart from the eminent gentamycin, ZnONPs (Table 7) have the highest zone of inhibition, indicating that it is the most effective treatment against bacteria [49]. Other studies have also investigated that ZnO nanoparticles are an effective agent for antibacterial activity because of their varied particle sizes and high surface area-to-volume ratios [50]. However, in a related vein, it has been discovered that the ZnO-NPs’ and Zn antibacterial action against different bacteria rises as the size of the particles decreases [46,51]. Although this study found that the susceptibility rate of the isolates studied, such as *Legionella and E. faecalis,* was zero, it was typically indicated that no activity had been shown. They have a history of antibiotic resistance, confirmed by the result of [48]. Also, *O. europaea* extract may decrease microbial respiration and promote plasma membrane permeability, killing bacterial cells after prolonged contact [52].

**Table 7:** Shows the disc diffusion result for the antibacterial activity of *O. europaea*, ZnONPs, Zn, gentamycin, H_2_O, and DMSO.

Bacteria	Gentamycin(mm)	ZnONPs(mm)	Zn(mm)	*O. europaea*(mm) (10 µL)	DMSO (mm)	Distilled Water(mm)
*B. Cereus*	0.0 ± 0.0	8.44 ± 1.22	8.0 ± .0.814	7.77 ± 0.56	0.0 ± 0.0	0.0 ± 0.0
*S. aureus*	36.96 ± 0.740	16.403 ± 1.233	7.99 ± .0.542	0.0 ± 0.0	0.0 ± 0.0	0.0 ± 0.0
*P. aeruginosa*	42.55 ± 1.796	29.22 ± 0.831	18.99 ± 1.512	0.0 ± 0.0	0.0 ± 0.0	0.0 ± 0.0
*E.coli*	35.77 ± 0.682	12.55 ± 2.313	8.22 ± 1.662	0.0 ± 0.0	0.0 ± 0.0	0.0 ± 0.0

### The minimum inhibition concentration (MIC)

**Fig 14:**
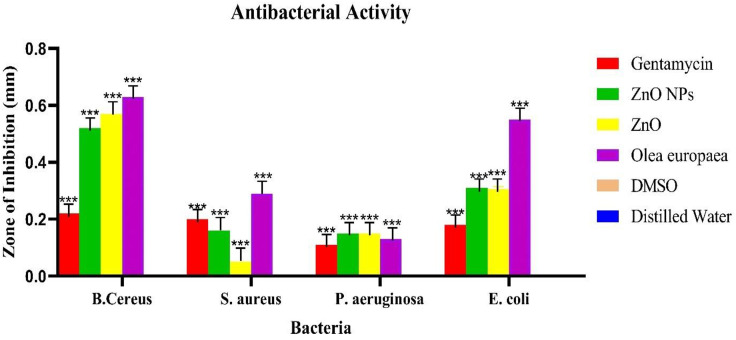
The minimum inhibition concentration of antibacterial agents like *O. europaea*, ZnONPs, ZnO, L-Gentamicin, H₂O, and DMSO against *B. cereus, S. aureus*, *P. aeruginosa*, and *E. coli.* The values are expressed as mean ± SD values and analyzed by two-way analysis of variance (ANOVA). An asterisk (_***_) indicates a significant difference among treatments concerning control (P < 0.0001).

**Fig 15:**
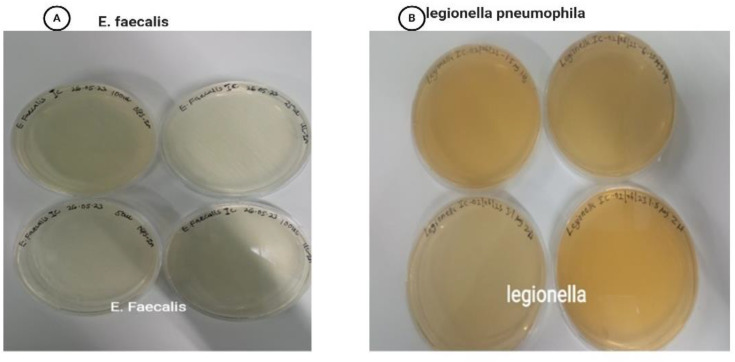
MBC Antibacterial activity of *O. europaea*, ZnONPs, and Zn *on* (A) *Legionella* and (B) *E. faecalis bacteria.*

For the MIC, the microdilution method with different concentrations was determined for *Legionella pneumophila, E. coli, S. aureus, B. cereus, E. faecalis, and P. aeruginosa* (Fig 14). The different concentrations of *O. europaea*, ZnO-NPs, and Zn inhibiting were visible. For the MIC, concentrations which are 100, 50, 25, 12.5, 6.25, 3.13, and 1.5 µL, in each bacterium, there were minimum inhibition concentrations, ZnONPs, Zn, *O. europaea* 1.5, 3.13, and 6.25 µL, *E. coli*, *S. aureus*, *B. cereus, E. faecalis*, and *P. aeruginosa*. *E. faecalis* and *Legionella pneumophila* after overnight incubation of the six bacterial cultures at 37°C (Fig 14) of various bacteria. Gentamycin has been the most effective, followed by ZnONPs, the ZnO precursor, and *O. europaea,* while DMSO and distilled water all show no zone of inhibition. The minimum inhibition concentration (MIC) results in antibacterial activity against *O. europaea*, ZnONPs, ZnO, L Gentamicin, H₂O, and DMSO. Gentamicin, ZnONPs, ZnO *O. europaea* DMSO, and distilled water demonstrated robust antimicrobial activity against all *Legionella pneumophila, E. coli, S. aureus, B. cereus, E. faecalis, and P. aeruginosa, as* indicated in the table below (Table 8). The results indicate that the ethanolic extract of *O. europaea* exhibited higher antibacterial sensitivity, particularly against B. cereus and E. coli, compared with both ZnO nanoparticles and bulk ZnO. This activity may be attributed to the presence of polyphenols, flavonoids, and triterpenoids in the plant extract. Following the MIC determination, broths were subcultured onto new agar plates to determine the MBC, which has the lowest *O. europaea*, ZnONPs, and Zn concentration, which should kill 99.9% of the bacteria. The MBC was used against *P. aeruginosa, B. cereus, E. coli, and S. aureus;* however, only E.* faecalis* and *Legionella pneumophila* have shown growth despite the treatment. Colony count was used to determine the bacterial density after the assay was performed (Fig 15, A and B) [20,48].

**Table 8:** Shows the MIC analysis of the different bacteria with the treatment agents.

Bacteria	Gentamycin (mm)	ZnONPs (mm)	ZnO (mm)	Olea (mm)	DMSO (mm)
*B. Cereus*	0.22 ± 0.033	0.523 ± 0.0052	0.572 ± 0.012	0.632 ± 0.073	0.508 ± 0.036
*S. aureus*	0.207 ± 0.0	0.165 ± 0.004	0.052 ± 0.01	0.293 ± 0.004	0.227 ± 0.0
*P. aeruginosa*	0.11 ± 0.0016	0.150 ± 0.0286	0.152 ± 0.0272	0.135 ± 0.0365	0.124 ± 0.0221
*E. coli*	0.188 ± 0.0	0.311 ± 0.0484	0.299 ± 0.0425	0.55 ± 0.0389	0.543 ± 0.0

### *In vitro* cytotoxicity and Antiproliferative activity Assay

**Fig 16:**
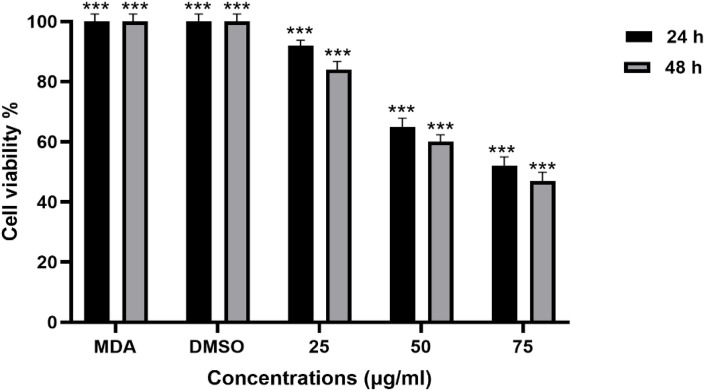
The graph shows the percentage of viable cells *in vitro* cytotoxic effect of ZnONPs on MDA-MB-231 breast cancer cell lines upon exposure to various concentrations of A1 (25 μg/mL), A2 (50 μg/mL) and A3 (75 μg/mL) in both 24 and 48 hours against the controls (super control) and (DMSO). The morphology of the MDA-MB-231 cell line was treated with ZnO nanoparticles, and the control was with concentrations of 25, 50, and 75 μg/mL for 24 and 48 hours. The bar chart reveals the cell viability percentage on the Y-axis against the plant extract concentration on the X-axis at different time points. The experiment was repeated three times, as shown by the error bar.

**Fig 17:**
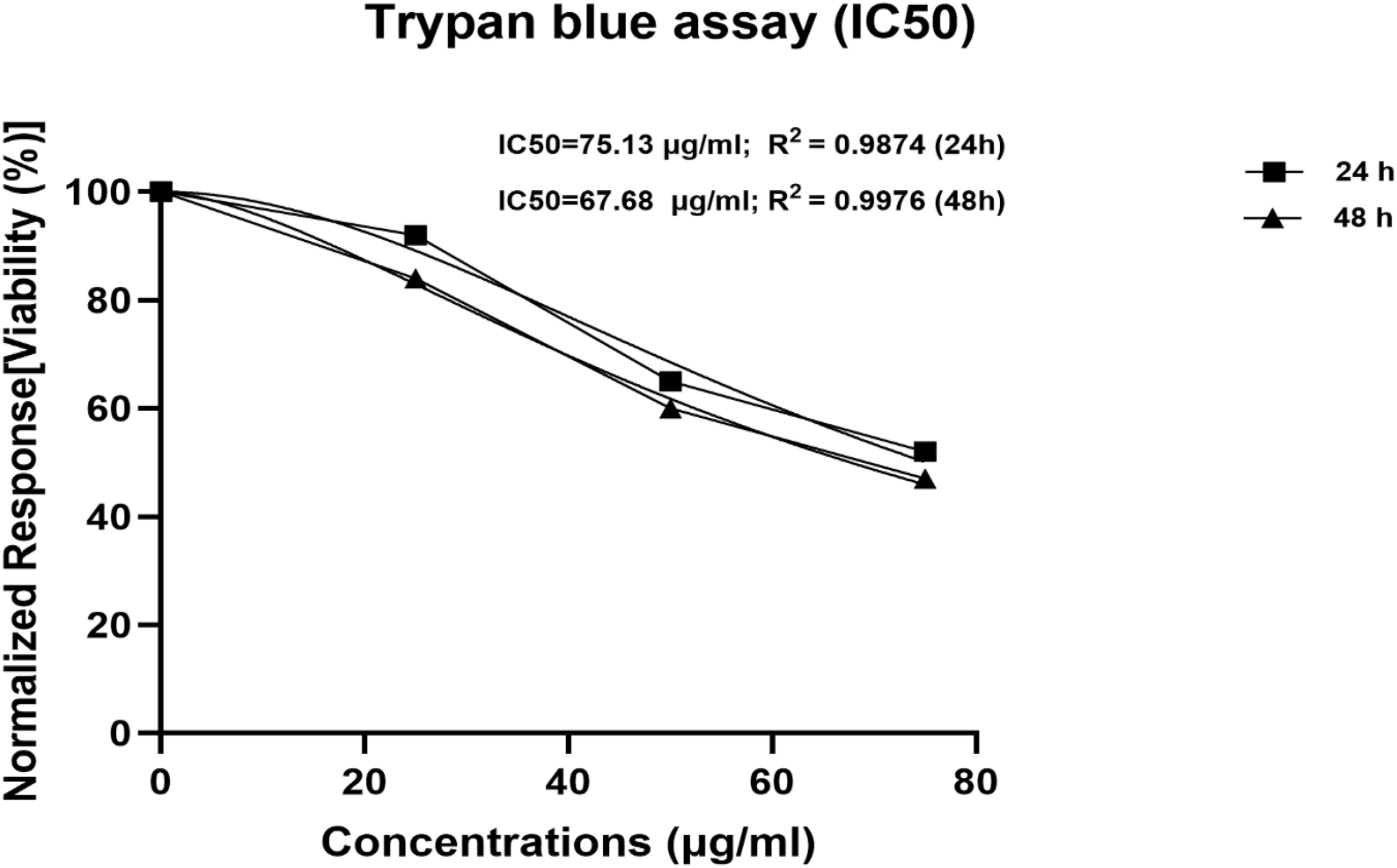
IC_50_ determined by nonlinear regression [curve fit (inhibitor) vs. normalized response–variable slope].

**Fig 18:**
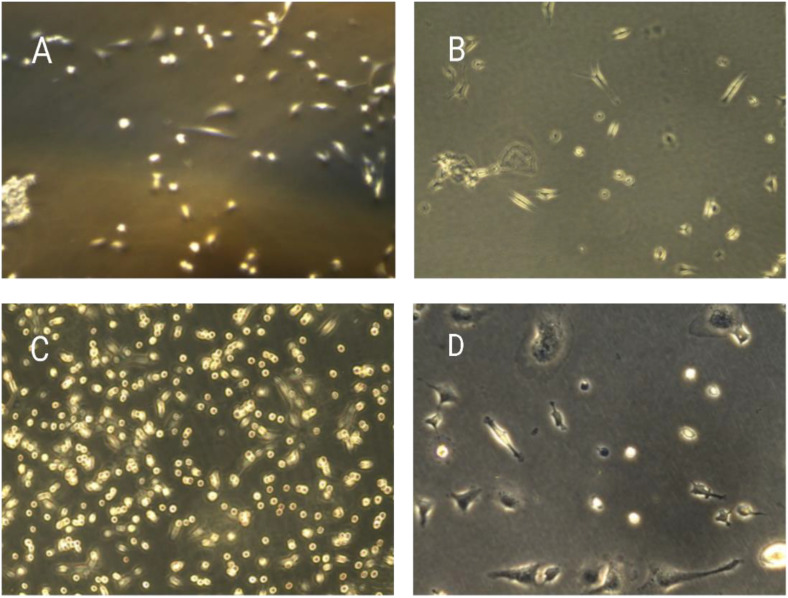
The morphologies of the control MDA-MB-231 breast cancer cells under the treatment. The graph shows the morphology of MDA-MB 231 breast cancer cell lines (A) and (C), which are the control, while (B) and (D) are cells that were treated with different concentrations with ZnONPs for 24 hours with 25 μg/mL, 50 μg/mL And 75 μg/mL which indicated a sign of apoptosis, with a more significant difference in dose-dependence.

**Fig 19:**
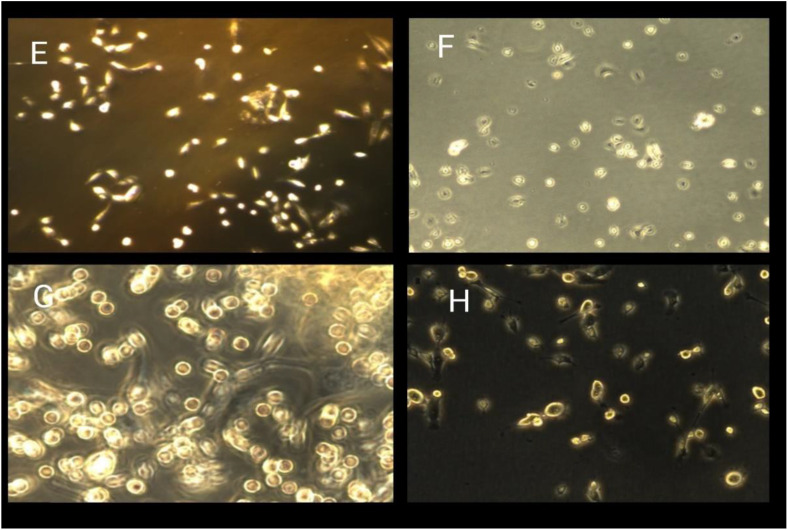
The morphology of the MDA-MB-231 breast cancer cells of the control group against the treatment. The morphology of MDA-MB 231 breast cancer cell lines (E) and (G) as controls, while (F) and (H) are cells that were treated with different concentrations of ZnONPs for 48 hours with 25 μg/mL, 50 μg/mL, and 75 μg/mL which indicated a sign of apoptosis, with a more significant difference in dose-dependence.

**Fig 20:**
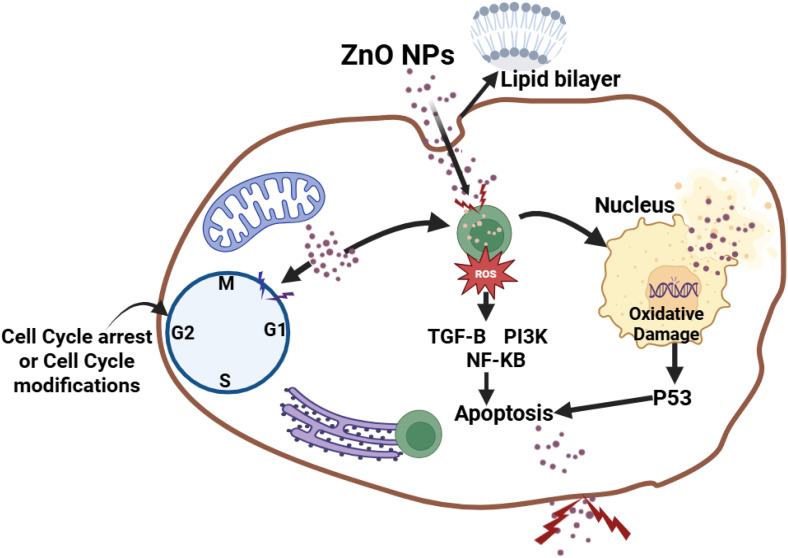
Proposed mechanism of the cytotoxicity activity of ZnONPs on MDA-MB-231 cancer cell growth.

The trypan blue assay indicated the viability of the breast cancer cells upon exposure to various concentration treatments used in both the 24-hour and 48-hour assays, respectively. The breast cancer cell lines MDA-MB-231 were tested for proliferation and the cytotoxic effects of ZnO nanoparticles. The ZnONPs (Figs 16 and 17) are said to be active against MDA-MB-231 cells after proliferation. At the end of 24 hours of incubation, a trypan blue exclusion experiment was performed to determine the effects of three different concentrations of ZnONPs (25, 50, and 75 μg/mL) (Fig 16) on the viability of MDA-MB-231 cell lines. The result from the controls (Fig 16A and C) showed the cells to have a viability rate of 100%. Meanwhile, by the end of 24 hours of treatment, the total number of cells had fallen to 60% (Fig 16 B and D), suggesting that the concentration of dosage had a cytotoxic effect on the cells, thereby leading to their death and decrease from 100% to 60%, and this continued to about 47% (Fig 17 F and H) after 48 hours of trypan blue application. More cell death occurred at 48 hours of treatment at 47% (Fig 17 F and H). The result indicates that ZnO nanoparticles have a cytotoxic effect on the MDA-MB-231 breast cancer cell line, as they show apoptosis behavior, including morphological alterations such as cell shrinkage and clumping, deformed cells, and suppression of cell growth [53]. Finally, by analyzing the observed results, the 25, 50, and 75 μg/mL concentrations of ZnONPs affected the breast cancer cells. Notably, there is a significant difference between the control group and the treatment group of ZnONPs on the MDA-MB-231 in the dosage-dependent amount and the time from the result, similar to the finding from [40]. The IC_50_ value indicates the concentration of ZnONPs needed to reduce 50% of the cancer cells (Figs 16 and 17). The values show the efficacy in stopping the MDA-MB-231 cancer cell growth. After the exposure of cells for 24 hours, a concentration of 75 μg/mL of ZnONPs causes a reduction of 50% in MDA-MB-231 cell viability. After 48 hours, a lower concentration of 50 μg/mL was needed to achieve 50% inhibition compared to the first dose administered; this determines the sensitivity of cells upon exposure to the nanoparticles for a longer period. This indicates that the 50 μg/mL has a cytotoxic effect on the ZnONPs when exposed to a longer period. Furthermore, the IC_50_ value (Fig 17) indicates a consistent reduction over time from 24 hours (75 μg/mL) to reach (50 μg/mL) in 48 hours. This finding suggests that the ZnONPs can kill or slow the MDA-MB-231 cancer cells’ growth over time (Fig 18 and Fig 19) [54,55]. Nanoparticles play a crucial role in signaling cascades where the signaling pathways for STAT3, β-catenin, ROS, NF-κB, Erk, Akt, Wnt, MPK, and PI3K cause DNA damage and apoptosis in addition to increasing oxidative stress (Fig 20). Through oxidative stress and mitochondrial membrane damage, the NF-κB pathway generates free radicals, triggering apoptosis and activating pro-inflammatory mediators. In many cancer types, PI3K/AKT is one of the intracellular pathways that is most activated because it controls angiogenesis and transcription while promoting metabolic reprogramming, cancer development, and survival (Fig 20). In both *in vitro* and *in vivo* models, applying nanoparticles via various natural processes has demonstrated an anticancer effect [19].

The morphology of the MDA-MB-231 cell line was treated with ZnO nanoparticles, and the control was with the concentration of 25, 50, and 75 μg/mL for 24 hours. The Apoptosis Morphology of Breast Cancer

The morphology of the MDA-MB-231 cell line was treated with ZnO nanoparticles, and the control was with the concentration of 25, 50, and 75 μg/mL for 48hours.

### Limitations and Future Perspectives of the Study

One of the most important aspects of the study is conducting *in vivo* validation studies. The *in vitro* assays provide foundational insights into cytotoxic activity; however, they are limited in explaining the complex mechanism of the tumor microenvironment and the pathways in which all the mechanisms of action take place. We will look at using animal models in the future, such as mice that are immunocompromised, and MDA-MB-231 cells will be implanted in them, and they will be treated with the extract *in vivo* and the synthesized nanoparticles so that observations can be made from their behavior. This type of approach will help us understand whether the plant extract can effectively suppress tumor growth or metastasis over time. Another important future perspective of the study is the isolation and structural characterization of the specific bioactive compound(s) responsible for the observed cytotoxic activity. *O. europaea* is known to contain numerous polyphenols and other bioactive compounds. However, there is a limitation in understanding which of the compounds is responsible for the cytotoxic effect caused to cancer cell lines. Because of this limitation, techniques such as bioassay-guided fractionation, high-performance liquid chromatography (HPLC), mass spectrometry (MS), and nuclear magnetic resonance (NMR) spectroscopy will be employed to isolate and identify the active compounds responsible for the cytotoxic effect. Thirdly, to get a better understanding of the mechanism of action of *O. europaea* extract, mechanistic studies will be performed, most especially in understanding the apoptotic pathway of action of the extract. The results from techniques such as flow cytometry, Western blotting, and transcriptome profiling will help in understanding whether the cytotoxic effect is due to apoptosis induction or inhibition of cell proliferation. Lastly, the scope of the study will be expanded to include other breast cancer subtypes to assess the broader applicability of the extract and also because of the limitation due to the availability of fibroblast cell lines, which in the next study will be used as a control for all cancer studies for proper observation and validation of the assay. Also, applying the extract to cancerous and non-cancerous cell lines to observe their behavior.

## Conclusion

The green synthesis of ZnO nanoparticles (ZnONPs) using *O. europaea* extract was determined through multiple analyses. UV-Vis spectroscopy showed peaks at 238, 282, and 313 nm, indicating nanoparticle formation. The zeta potential of −12.64 mV and particle size of 86 nm analyses suggested good colloidal stability. GC-MS identified bioactive compounds in the extract, while XRD suggested crystallinity, and FTIR revealed functional groups involved in nanoparticle stabilization. SEM and SEM-EDS analyses confirmed spherical morphology and elemental composition of ZnONPs. Antioxidant (DPPH) assays demonstrated free radical scavenging activity of the synthesized extract. The antimicrobial tests showed inhibition zones against various pathogenic bacteria. Furthermore, cytotoxicity assays on MDA-MB-231 breast cancer cells indicated a time-dose-dependent reduction in cell viability. Overall, *O. europaea*-mediated ZnONPs exhibit promising multifunctional bioactivities, including antioxidant, antimicrobial, and anticancer properties, suggesting their potential for biomedical and therapeutic applications.
